# Tea Drinking and Its Association with Active Tuberculosis Incidence among Middle-Aged and Elderly Adults: The Singapore Chinese Health Study

**DOI:** 10.3390/nu9060544

**Published:** 2017-05-25

**Authors:** Avril Zixin Soh, An Pan, Cynthia Bin Eng Chee, Yee-Tang Wang, Jian-Min Yuan, Woon-Puay Koh

**Affiliations:** 1 Saw Swee Hock School of Public Health, National University of Singapore, Singapore 117549, Singapore; avril.soh@u.nus.edu; 2 Department of Epidemiology and Biostatistics, Ministry of Education Key Laboratory of Environment and Health, and State Key Laboratory of Environmental Health (incubation), School of Public Health, Tongji Medical College, Huazhong University of Science and Technology, Wuhan 430030, China; panan@hust.edu.cn; 3 Singapore Tuberculosis Control Unit, Tan Tock Seng Hospital, Singapore 308089, Singapore; cynthia_chee@ttsh.com.sg (C.B.E.C.); yee_tang_wang@ttsh.com.sg (Y.-T.W.); 4 Division of Cancer Control and Population Sciences, University of Pittsburgh Cancer Institute, and Department of Epidemiology, University of Pittsburgh Graduate School of Public Health, Pittsburgh, PA 15261, USA; yuanj@upmc.edu; 5 Office of Clinical Sciences, Duke-NUS Medical School, 8 College Road, Singapore 169857, Singapore

**Keywords:** tea, tuberculosis, epidemiology

## Abstract

Experimental studies showed that tea polyphenols may inhibit growth of *Mycobacterium tuberculosis*. However, no prospective epidemiologic study has investigated tea drinking and the risk of active tuberculosis. We investigated this association in the Singapore Chinese Health Study, a prospective population-based cohort of 63,257 Chinese aged 45–74 years recruited between 1993 and 1998 in Singapore. Information on habitual drinking of tea (including black and green tea) and coffee was collected via structured questionnaires. Incident cases of active tuberculosis were identified via linkage with the nationwide tuberculosis registry up to 31 December 2014. Cox proportional hazard models were used to estimate the relation of tea and coffee consumption with tuberculosis risk. Over a mean 16.8 years of follow-up, we identified 1249 incident cases of active tuberculosis. Drinking either black or green tea was associated with a dose-dependent reduction in tuberculosis risk. Compared to non-drinkers, the hazard ratio (HR) (95% confidence interval (CI)) was 1.01 (0.85–1.21) in monthly tea drinkers, 0.84 (0.73–0.98) in weekly drinkers, and 0.82 (0.71–0.96) in daily drinkers (*p* for trend = 0.003). Coffee or caffeine intake was not significantly associated with tuberculosis risk. In conclusion, regular tea drinking was associated with a reduced risk of active tuberculosis.

## 1. Introduction

Tuberculosis is caused by infection with *Mycobacterium tuberculosis* (Mtb), and approximately 5–15% of the estimated 2–3 billion infected individuals in the world will develop active tuberculosis during their lifetime [1]. With the very large reservoir of latently-infected individuals, understanding the factors affecting reactivation of latent tuberculosis infection is imperative in preventing new cases of active tuberculosis. 

During latent tuberculosis infection, a continuum of immunologic responses are encompassed within the dynamic balance between the host and pathogen, and active disease occurs when bacterial replication exceeds host protective responses [2,3]. One of the host-defense mechanisms during tuberculosis infection include the production of reactive intermediates against Mtb [4], and this generation of free radicals in excess of the host antioxidant capacity leads to oxidative stress [5]. Furthermore, progressive oxidative stress during experimental tuberculosis in guinea pigs has been shown to be partially restored with antioxidant treatment, suggesting that the therapeutic strategies that reduce oxidant-mediated tissue damage may be beneficial as an adjunct therapy in the treatment and prevention of tuberculosis in humans [6]. 

Tea is a widely consumed beverage worldwide. Both black tea and green tea exhibit antioxidant properties [7,8], and many experimental studies have shown tea polyphenols to be beneficial against many diseases by ameliorating levels of oxidative stress [9,10,11,12]. Existing literature investigating the effect of tea polyphenols on tuberculosis has mainly focused on green tea catechins. Green tea extract has been reported to reduce oxidative stress associated with tuberculosis in infected mice in an experimental study [13], and in tuberculosis patients in a clinical study [14]. Epigallocatechin gallate (EGCG), the major component of green tea catechins, has also been shown experimentally to inhibit mycobacterial survival [15,16,17]. Epidemiological evidence to support the role of tea drinking in the development of tuberculosis is scarce; only one recent case-control study has reported tea drinking to be inversely associated with prevalent tuberculosis [18]. However, the temporal association between tea drinking and tuberculosis remains unclear.

In this study, we investigated the prospective relation between tea drinking and risk of developing active tuberculosis in a population-based cohort in Singapore. Participants of this cohort went through periods when tuberculosis was highly prevalent in the country a few decades ago, and those who acquired latent tuberculosis infection in those early years would be at risk of disease reactivation at advanced age [19]. 

## 2. Materials and Methods 

### 2.1. Study Population

A total of 63,257 Chinese adults (27,959 men and 35,298 women) were enrolled in the Singapore Chinese Health Study between 1993 and 1998 [20], and the inclusion criteria was based on the participant’s age, dialect group, and residency status. The recruited study participants were aged 45–74 years at recruitment, and were restricted to the two major dialect groups in Singapore: the Hokkiens who came from Fujian Province, and the Cantonese who came from Guangdong province in China. The cohort included citizens or permanent residents of Singapore living in government-built housing estates, where 86% of the Singapore population resided during the period of recruitment [20]. Recruitment was initiated using posted letters to invite residents from public housing estates to take part in the study. Interviewers went door-to-door 5–7 days later to recruit participants for the study if they met the inclusion criteria based on their ethnicity, age, dialect group, and residency status. Approximately 85% of the eligible subjects invited agreed to participate [21]. This study was approved by the Institutional Review Board at the National University of Singapore in September 2011 (approval number NUS-1396), and all participants gave informed consent. 

### 2.2. Assessment of Tea Intake and Other Covariates at Baseline

At recruitment, a face-to-face interview was conducted using a structured questionnaire, and information collected included participant demographics, height, weight, lifetime use of tobacco, alcohol consumption, and history of physician-diagnosed medical conditions, such as diabetes and cancer. Body mass index (BMI) of each participant was calculated with the use of the formula: weight (kg)/height (m)^2^. A 165-item semi-quantitative food-frequency questionnaire (FFQ) specifically developed and validated for this study population was used to assess the participant’s usual diet over the past year [20]. For the intake frequency of black tea, green tea, and coffee, participants were asked to choose from nine predefined categories (never or hardly ever, 1–3 cups/month, 1 cup/week, 2–3 cups/week, 4–6 cups/week, 1 cup/day, 2–3 cups/day, 4–5 cups/day, and 6 or more cups/day). Oolong, a semi-fermented tea, was grouped together with green tea in the FFQ since it was drunk interchangeably with green tea in our study population. Caffeine intake was estimated from the participant’s reported intake of tea and coffee. The main sources of caffeine in this study population include coffee (82%), black tea (13%), and green tea (<5%) [22]. 

### 2.3. Ascertainment of Tuberculosis Cases 

Cases of active tuberculosis were identified via linkage with the National Tuberculosis Notification Registry, which was started in 1957 [23]. Notification of tuberculosis cases is compulsory under the Infectious Diseases Act in Singapore [24], and all doctors are mandated by law to notify all suspected and confirmed cases of tuberculosis to the Ministry of Health within 72 h. Most of the tuberculosis cases in Singapore are diagnosed by passive case-finding when patients present with symptoms, such as persistent cough, blood-stained sputum, fever and chills, and night sweats. A case of active tuberculosis is diagnosed by positive sputum smear and confirmed by culture tests. The principal sources of notification are the restructured public hospitals and the Tuberculosis Control Unit, accounting for 56% and 34% of notifications, respectively. In addition to notification by doctors, all culture-positive tuberculosis patients in Singapore are also captured comprehensively in the National Tuberculosis Notification Registry via electronic linkage with the two mycobacterial laboratories in Singapore [23]. 

The cohort was also actively followed by regular linkage to the Singapore Registry of Births and Deaths to update vital status of the cohort members. As of 31 December 2014, only 52 participants were known to be lost to follow-up due to migration out of Singapore or other reasons. 

### 2.4. Statistical Analysis

We excluded participants with a history of active tuberculosis before recruitment (*n* = 3012), identified through linkage with the National Tuberculosis Notification Registry. The final analysis included 60,245 participants (Supplementary Figure S1). The baseline characteristics of the participants by their frequency of tea intake were compared using analysis of variance (ANOVA) [25] for continuous variables and chi-square test [26] for categorical variables. For each participant, person-years were calculated from the date of recruitment to date of diagnosis of tuberculosis, death, lost-to-follow-up, or 31 December 2014, whichever occurred earlier. Participants were categorized based on their intake frequency of tea and coffee into non-drinkers (less than monthly), monthly, weekly, or daily drinkers. Cox proportional hazard regression models [27] were used to assess the associations between intake frequency of tea and coffee, and quartile intake of caffeine and tuberculosis risk. The strength of an association was measured by the hazard ratio (HR) and its corresponding 95% confidence interval (CI). *p*-values for trend were computed via the likelihood ratio test by using the ordinal values of intake categories for tea and coffee, or quartile intake of caffeine as continuous variables in the Cox regression models. There was no violation of Cox proportional hazard assumptions for our variables of interest.

The model was first adjusted for age at recruitment (years), year of recruitment (1993–1995, 1995–1998), gender, and dialect group (Hokkien, Cantonese). Additionally, we adjusted for the level of education (no formal education, primary school, secondary school, or higher), BMI (kg/m^2^, continuous), baseline history of diabetes (yes, no), smoking status and intensity (never, former 1–12 cig/day, former 13–22 cig/day, former 23+ cig/day, current 1–12 cig/day, current 13–22 cig/day, current 23+ cig/day), and alcohol consumption (none, monthly, weekly, daily). These factors have been shown to affect tuberculosis risk either in the literature [28] or in our cohort (Supplementary Table S3) and could be potential confounders. Finally, we adjusted for the intake of green tea, black tea, and coffee concurrently. 

We also explored the interaction between tea drinking and other established factors of tuberculosis, such as age, gender, BMI categories, smoking status, alcohol consumption, and baseline history of diabetes. The classification of BMI levels was based on categories recommended for potential public health action points for Asian populations by the World Health Organization [29]. The heterogeneity of the tea-tuberculosis associations by different factors was tested using an interaction term (product between tea drinking categories and factor of interest) in the Cox model. Finally, to overcome the possibility of reverse-causality bias, we repeated our analysis by excluding tuberculosis cases diagnosed within two years post-enrollment and the corresponding observed person-years.

All statistical analyses were conducted using SAS version 9.3 (SAS Institute, Cary, NC, USA) statistical software package. Two-sided *p*-value < 0.05 was considered statistically significant.

## 3. Results

In this cohort, 41.3% of participants were non-drinkers of tea (defined as drinking less than 1 cup per month), and 22.3% were daily tea drinkers (Table 1). Among daily tea drinkers, 40.0% drank only black tea, 46.4% drank only green tea, and the remaining 13.6% were not exclusive in the type of tea they consumed. Among daily black tea drinkers, 19.1% also drank green tea daily, and among daily green tea drinkers, 17.0% also drank black tea daily (Supplementary Table S1). Compared with non-drinkers, daily tea drinkers were more likely to be men, former or current smokers, have higher level of education, consume alcohol and report history of diabetes at baseline (Table 1). Compared to daily green tea drinkers, daily black tea drinkers were younger, had a lower prevalence of diabetes at baseline, and were more likely to be men, Hokkiens, and current smokers (Supplementary Table S1). Tuberculosis cases were also more likely to be older, men, smokers, have a history of diabetes at baseline, lower BMI, received lower level of education, and have a higher intake frequency of alcohol compared to participants who did not develop active tuberculosis (Supplementary Table S2). 

Over a mean 16.8 ± 5.2 years of follow-up, we identified 1249 incident cases of active tuberculosis. The incidence rates of tuberculosis within this cohort, adjusted to the age structure of the whole cohort, were 224 per 100,000 person-years in men and 55 per 100,000 person-years in women. For tuberculosis cases, the mean duration from time of recruitment to tuberculosis diagnosis was 8.9 ± 5.6 years, and mean age at diagnosis was 68.7 ± 9.1 years. We observed an inverse association between the consumption of both black and green tea with tuberculosis risk in a dose-dependent manner (Table 2). Compared to non-drinkers of the particular type of tea, a reduced risk of tuberculosis was observed among participants who had a daily consumption of black tea (HR 0.79; 95% CI 0.65–0.95; *p* for trend = 0.02) or green tea (HR 0.84; 95% CI 0.70–1.00; *p* for trend = 0.03). Compared to non-drinkers of tea, drinking either black or green tea daily reduced risk of tuberculosis (HR 0.82; 95% CI 0.71–0.96; *p* for trend = 0.003). There was no significant association between coffee and caffeine intake and tuberculosis risk (Table 2). 

We did not observe any statistically significant modification of the tea-tuberculosis association with age, gender, smoking status, or baseline history of diabetes (*p* for interaction > 0.20; data not shown). A significant interaction between tea drinking and BMI categories was observed. Daily tea drinking was associated with reduced tuberculosis risk among leaner participants with BMI < 23 kg/m^2^ (HR 0.66; 95% CI 0.54–0.81), but was not among participants with higher BMI (HR 1.09; 95% CI 0.87–1.36; *p* for interaction = 0.004). There was also significant interaction between tea drinking and alcohol consumption. A greater reduction in tuberculosis risk with daily tea drinking was observed among weekly/daily alcohol drinkers (HR 0.54; 95% CI 0.38–0.77) compared to participants with less regular alcohol consumption (HR 0.90; 95% CI 0.77–1.06; *p* for interaction = 0.01) (Table 3).

The association between tea drinking and tuberculosis risk remained essentially the same after exclusion of participants diagnosed with tuberculosis within the first two years after their recruitment (data not shown). We also examined the associations among participants who were exclusive drinkers of either black or green tea. Compared to non-drinkers, participants who only drank black or green tea daily had similarly reduced risk of tuberculosis; HR (95% CI) were 0.79 (0.63–0.98) for daily drinkers of black tea only and 0.87 (0.71–1.06) for daily drinkers of green tea only. Comparatively, daily tea drinkers who drank both types of tea had the lowest reduced risk of 0.73 (95% CI: 0.56–0.95). We have also additionally adjusted for dietary factors such as fruits and vegetables intake in the model, and the results remained essentially unchanged (data not shown).

## 4. Discussion

To our best knowledge, this is the first prospective study investigating the relation between tea drinking and tuberculosis risk, and we found that drinking tea (either black or green tea) was inversely associated with risk of developing active tuberculosis in a dose-dependent manner. The reduction in tuberculosis risk with daily tea drinking was greater in leaner participants (BMI < 23 kg/m^2^) relative to their overweight/obese counterparts, as well as in weekly/daily alcohol drinkers compared to participants who drank alcohol less frequently. No association was observed with coffee or caffeine consumption. 

Epidemiologic evidence on the relation between tea drinking and tuberculosis risk is limited. Only one recent case-control study reported a possible inverse association with consumption of ≥1 cup/week of black tea (odds ratio 0.68; 95% CI 0.52–0.90) or green tea (odds ratio 0.53; 95% CI 0.35–0.82) [18], which was consistent with the findings of the present study. 

Both black and green teas are produced from the leaves of *Camellia sinensis*, and they vary in their polyphenol content due to different manufacturing processes. Green tea is made by drying fresh tea leaves without prior fermentation, and this preserves the naturally occurring polyphenols, such as tea catechins [30]. The production of black tea, on the other hand, involves the crushing of fresh tea leaves, leading to fermentation and oxidation of tea catechins into other polyphenols, such as theaflavins [30]. To date, experimental investigations of tea polyphenols on tuberculosis have only involved green tea catechins. EGCG, the most abundant catechin present in green tea, has been shown to have anti-mycobacterial effects by inhibiting Mtb enoyl-acyl reductase (InhA), an enzyme involved in the production of functional mycolic acids [16]. Structural damage in the mycobacterial cell wall due to impaired production of mycolic acids has been postulated as a mechanism behind the anti-mycobacterial function of EGCG [15]. In experimental studies, mice infected with Mtb had significantly increased levels of oxidative stress during early stages of tuberculosis, and oral administration of green tea extract led to the reversion of oxidative stress parameters to near normal levels [13]. Pre-treatment of macrophages with 60 µg/mL (≈131 µmol/L) of EGCG has been shown to lead to down-regulation of the expression of the host molecule tryptophan-aspartate containing coat protein (TACO), and resultant inhibition of mycobacterium survival within macrophages [17]. The highest tea consumers in our cohort reported intake of six or more cups of black or green tea per day, and the consumption of six cups of black or green tea has been shown to increase blood catechin levels with an average maximum change from baseline by 0.10 µmol/L and 0.46 µmol/L respectively [31]. While the increase in blood catechin levels with tea consumption in human is much lower than what has been achieved in experimental settings using animal models, the increased levels of catechins with tea consumption has been linked to increased plasma antioxidant activity and possible protection against diseases [31,32,33]. Even though the effect of black tea polyphenols on tuberculosis infection or disease has not been investigated in experimental studies, black tea polyphenols, such as theaflavins have been shown to exhibit similar antioxidant potency as green tea catechins [7,34]. These experimental findings suggest the potential of tea polyphenols against development of active tuberculosis, and support the observations from epidemiological studies. 

We attempted to understand the biological mechanism of tea in reducing tuberculosis risk by examining how other established risk factors of tuberculosis may modify this effect. We found daily tea drinking to have a more prominent effect in leaner individuals compared to obese/overweight individuals. Leanness has been thought to increase tuberculosis risk due to lower protein and energy intake among lean individuals [35]. However, this cannot explain the greater risk reduction with tea drinking among leaner participants in our study as tea does not contribute significantly to protein or energy intake. Furthermore, the estimates for the tea-tuberculosis association did not change when we included protein and total energy intake in the model (data not shown). Similarly, we found that daily tea drinking conferred a greater protective effect in weekly/daily alcohol drinkers compared to less regular alcohol drinkers. Although alcohol consumption can impair the immune system and increase tuberculosis risk [36,37,38,39], the meta-analysis by Lönnroth et al. concluded that a substantial increase in risk of developing active tuberculosis was only observed in individuals who consumed more than 40 g/day of alcohol, and/or had an alcohol use disorder, but not in people who drank less than 40 g/day [38]. Only 0.91% of the participants in our cohort reported an average alcohol intake of more than 40 g/day. On top of these plausible mechanisms, leanness and alcohol consumption are also factors associated with increased levels of oxidative stress [40,41]. Hence tea, a beverage rich in antioxidants, may confer a protective effect against tuberculosis, in part by ameliorating the increased levels of oxidative stress associated with leanness or alcohol consumption in affected individuals. Further studies are needed to evaluate possible anti-mycobacterial effects of polyphenols from black and green teas, and validate the effect of tea drinking in different subpopulations at risk of tuberculosis. 

Even though caffeine has been reported to have immunomodulatory effects [42], we did not observe any associations between caffeine intake and tuberculosis risk in our study. A significant relation between coffee and tuberculosis risk has never been reported, and we did not find any significant association with coffee in this study. The results provide indirect evidence that caffeine in tea is unlikely responsible for the inverse association between tea and tuberculosis.

Even though Singapore currently has an intermediate tuberculosis incidence rate of about 40 per 100,000 population, our study cohort consist of older residents of the country who were likely to be exposed to the bacteria during the 1960s, a period where incidence of tuberculosis was as high as 300 per 100,000 population [43]. Hence, many of our participants could have acquired latent infection in those early years where tuberculosis was far more rampant [19], and this makes our cohort suitable to study risk factors associated with tuberculosis reactivation. The observation of lower BMI, greater proportion of men, smokers and prevalent diabetes among tuberculosis cases compared to participants who remained free of tuberculosis in our cohort was also consistent with findings from the local population and other study populations [28,43]. Another strength of this study is the variability in intake frequencies of the two different types of tea in the population, which allowed us to simultaneously examine the relation of both black and green tea with tuberculosis risk in the same analysis. The prospective population-based design of our study also ensured minimal recall bias in exposure data since they were obtained years before tuberculosis diagnosis. By linking up with the national tuberculosis registry, in which notification of tuberculosis cases in the country is mandated by law, we were able to attain near-complete ascertainment of all diagnosed active tuberculosis cases in our cohort. 

Limitations of our study include using baseline intake of tea in our analysis, and changes to the frequency of tea consumption following the baseline interview were not accounted for. However, among 39,528 participants contacted for a follow-up interview between 2006 and 2010, an average of 12.7 years after the baseline interview, 85.6% of them retained their status as daily or non-daily drinkers of black tea, and 85.2% of them retained their status as daily or non-daily drinkers of green tea. This suggests the stability of tea drinking behaviour in our study population. Nevertheless, we acknowledge that any change in the habit of tea-drinking after recruitment could lead to potential non-differential misclassification of tea drinking, and underestimation of the true association. We were unable to determine whether green tea and semi-fermented oolong tea could have a different effect on tuberculosis risk as these two types of tea were included as a single inquiry in our FFQ. However, since black and green teas were both found to be associated with reduced active tuberculosis risk to a similar extent, we believe that drinking oolong tea would also have the same effect, and the grouping of oolong together with green tea is unlikely to influence the results of green tea. Our study is also limited to the analysis of late-life tuberculosis as our cohort consists of only middle-aged and elderly adults. Even though we did not adjust for the use of systemic immunosuppressant, a risk factor for tuberculosis, the use of systemic immunosuppressant that is sufficient to affect tuberculosis risk is unlikely to be high in a population-based cohort. We also lacked information on the participant’s human immunodeficiency virus status, which is also a risk factor for active tuberculosis. However, Singapore has relatively low human immunodeficiency virus infection rate in the general population [43]. We also lacked information on whether the participants had been infected with Mtb previously, and there could also have been undiagnosed cases of active tuberculosis that were not captured in the national tuberculosis notification registry. However, such cases are not expected to be high as medical and healthcare services in Singapore are generally affordable and efficient, and there is comprehensive capture of active tuberculosis cases mandated by the Singapore Infectious Diseases Act. Finally, as in any observational studies, causation could not be established, and residual confounding is still possible despite having adjusted for a number of confounding factors in the statistical models.

## 5. Conclusions

In conclusion, we observed an inverse association between tea consumption and tuberculosis risk in a prospective cohort of Chinese adults in Singapore. The protective association with tea drinking was more prominent among lean individuals and among weekly/daily alcohol drinkers. Since these individuals could be more susceptible to oxidative stress, it is conceivable that the antioxidant properties of tea polyphenols could be the biological driver underlying our observations, but this theory needs to be validated in future studies. Since both black and green teas are widely consumed worldwide, our findings have significant clinical and public health implications and tea drinking could potentially be used as a prophylactic measure against tuberculosis in susceptible populations. However, further research is essential to identify the bioactive compounds in black and green teas and to determine the mechanistic ways for the anti-mycobacterial effects in these compounds. 

## Figures and Tables

**Table 1:** Baseline characteristics of participants according to frequency of tea consumption ^1^.

Characteristics ^2^	Intake Frequency of Black or Green Tea			
	None	Monthly	Weekly	Daily
No. of participants (%)	24,859 (41.3)	7275 (12.1)	14,705 (24.4)	13,406 (22.3)
Age at interview, years	57.0 ± 8.1	56.3 ± 8.0	55.6 ± 7.9	56.1 ± 7.9
Body mass index, kg/m^2^	23.0 ± 3.2	23.2 ± 3.3	23.3 ± 3.3	23.5 ± 3.3
Men	8554 (34.4)	2766 (38.0)	6969 (47.4)	7625 (56.9)
Dialect				
Cantonese	10,641 (42.8)	3544 (48.7)	6892 (46.9)	6900 (51.5)
Hokkien	14,218 (57.2)	3731 (51.3)	7813 (53.1)	6506 (48.5)
Level of education				
No formal education	8796 (35.4)	2092 (28.8)	3261 (22.2)	2461 (18.4)
Primary school (1–6 years)	10,623 (42.7)	3280 (45.1)	6558 (44.6)	6055 (45.2)
Secondary school and above	5440 (21.9)	1903 (26.2)	4886 (33.2)	4890 (36.5)
Smoking status				
Never	18,046 (72.6)	5298 (72.8)	10,410 (70.8)	8678 (64.7)
Former	2253 (9.1)	739 (10.2)	1621 (11.0)	1824 (13.6)
Current	4560 (18.3)	1238 (17.0)	2674 (18.2)	2904 (21.7)
Alcohol intake				
None	21,288 (85.6)	5894 (81.0)	11,570 (78.7)	10,298 (76.8)
Monthly	1239 (5.0)	654 (9.0)	1291 (8.8)	1145 (8.5)
Weekly	1507 (6.1)	531 (7.3)	1398 (9.5)	1400 (10.4)
Daily	825 (3.3)	196 (2.7)	446 (3.0)	563 (4.2)
Baseline history of diabetes	2080 (8.4)	648 (8.9)	1329 (9.0)	1344 (10.0)

^1^ Data shown are *n* (%) for categorical variables and mean ± SD for continuous variables; ^2^ All *p*-values for differences in baseline characteristics of participants according to frequency of tea consumption by ANOVA (continuous variables) or chi-square test (categorical variables) were <0.001.

**Table 2:** Intake of tea, coffee, and caffeine in relation to risk of tuberculosis.

**Beverage**	**Intake Frequency**				***p* for Trend**
	**None**	**Monthly**	**Weekly**	**Daily**	
Black tea					
Person-years	646,006	78,649	174,715	113,319	
Cases	816	99	202	132	
HR (95% CI) ^1^	1.00	1.01 (0.82–1.25)	0.84 (0.72–0.98)	0.75 (0.63–0.91)	<0.001
HR (95% CI) ^2^	1.00	1.06 (0.86–1.31)	0.92 (0.78–1.07)	0.79 (0.66–0.95)	0.02
HR (95% CI) ^3^	1.00	1.10 (0.89–1.36)	0.94 (0.80–1.10)	0.79 (0.65–0.95)	0.02
Green tea					
Person-years	595,265	118,101	175,055	124,268	
Cases	764	130	200	155	
HR (95% CI) ^1^	1.00	0.86 (0.71–1.03)	0.83 (0.71–0.97)	0.78 (0.66–0.93)	<0.001
HR (95% CI) ^2^	1.00	0.90 (0.75–1.09)	0.89 (0.76–1.04)	0.84 (0.70–1.00)	0.02
HR (95% CI) ^3^	1.00	0.89 (0.74–1.08)	0.90 (0.77–1.06)	0.84 (0.70–1.00)	0.03
Black or green tea					
Person-years	415,819	122,737	248,903	225,232	
Cases	531	155	277	286	
HR (95% CI) ^1^	1.00	0.98 (0.82–1.17)	0.78 (0.67–0.90)	0.77 (0.66–0.89)	<0.001
HR (95% CI) ^2^	1.00	1.01 (0.85–1.21)	0.84 (0.73–0.98)	0.82 (0.71–0.96)	0.003
Coffee					
Person-years	187,395	19,633	93,401	712,261	
Cases	219	16	90	924	
HR (95% CI) ^1^	1.00	0.70 (0.42–1.16)	0.81 (0.63–1.03)	1.10 (0.95–1.28)	0.08
HR (95% CI) ^2^	1.00	0.72 (0.43–1.19)	0.84 (0.65–1.07)	0.97 (0.83–1.12)	0.89
HR (95% CI) ^3^	1.00	0.71 (0.43–1.18)	0.82 (0.64–1.06)	0.92 (0.79–1.08)	0.55
	**Quartile Intake**				
	**Q1**	**Q2**	**Q3**	**Q4**	
Caffeine (mg/day)					
Person-years	252,489	254,103	259,394	246,704	
Cases	205	305	316	373	
HR (95% CI) ^1^	1.00	1.20 (1.02–1.42)	1.06 (0.90–1.25)	1.20 (1.02–1.41)	0.10
HR (95% CI) ^2^	1.00	1.11 (0.94–1.32)	0.95 (0.80–1.12)	0.98 (0.83–1.16)	0.40

^1^ HR = hazard ratio, CI = confidence interval. Model 1 was adjusted for age at recruitment (years), year of recruitment (1993–1995, 1996–1998), gender, and dialect group (Hokkien, Cantonese); ^2^ Further adjusted for education level (no formal education, primary school, secondary school or higher), body mass index (kg/m^2^, continuous), baseline history of diabetes (yes, no), smoking status and intensity (never, former 1–12 cig/day, former 13–22 cig/day, former 23+ cig/day, current 1–12 cig/day, current 13–22 cig/day, current 23+ cig/day), alcohol intake (none, monthly, weekly, daily); ^3^ Further adjusted for intake of black and green tea and coffee (none, monthly, weekly, daily).

**Table 3:** Interaction between tea drinking and stratifying variables in relation to risk of tuberculosis.

Stratifying Variables	Intake Frequency of Black or Green Tea				*p* for Trend	*p* for Interaction
	None	Monthly	Weekly	Daily		
Body mass index						0.004
<23 kg/m^2^						
Cases	350	96	168	146		
HR (95% CI) ^1^	1.00	0.99 (0.79–1.25)	0.79 (0.66–0.96)	0.66 (0.54–0.81)	<0.001	
≥23 kg/m^2^						
Cases	181	59	109	140		
HR (95% CI) ^1^	1.00	1.05 (0.78–1.41)	0.92 (0.72–1.17)	1.09 (0.87–1.36)	0.70	
Alcohol intake						0.01
None/monthly						
Cases	432	137	225	242		
HR (95% CI) ^1^	1.00	1.10 (0.90–1.33)	0.86 (0.73–1.02)	0.90 (0.77–1.06)	0.08	
Weekly/daily						
Cases	99	18	52	44		
HR (95% CI) ^1^	1.00	0.62 (0.38–1.03)	0.71 (0.50–0.99)	0.54 (0.38–0.77)	<0.001	

^1^ HR = hazard ratio, CI = confidence interval. Model was adjusted for age at recruitment (years), year of recruitment (1993–1995, 1996–1998), gender, dialect group (Hokkien, Cantonese), education level (no formal education, primary school, secondary school or higher), body mass index (kg/m^2^, continuous), baseline history of diabetes (yes, no), smoking status and intensity (never, former 1–12 cig/day, former 13–22 cig/day, former 23+ cig/day, current 1–12 cig/day, current 13–22 cig/day, current 23+ cig/day), alcohol intake (none, monthly, weekly, daily).

## Supplementary Materials

The following are available online at www.mdpi.com/2072-6643/9/5/544/s1, Figure S1: Flow diagram of participants included in the final analysis, Table S1: Baseline characteristics of daily tea drinkers by different types of tea, Table S2: Baseline characteristics of participants who developed tuberculosis (TB) and those who remained free of TB, Table S3: Baseline factors in relation to risk of active tuberculosis.
