# Supplementary material for: Tea Drinking and Its Association with Active Tuberculosis Incidence among Middle-Aged and Elderly Adults: The Singapore Chinese Health Study
Source: Nutrients. 2017 May 25;9(6):544. doi: 10.3390/nu9060544 (PMC5490523; doi:10.3390/nu9060544)
Supplement: Supplementary file 1 [file nutrients-09-00544-s001.pdf]

Tea Drinking and Its Association with Active Tuberculosis Incidence among Middle-Aged and Elderly Adults: The Singapore Chinese Health Study

Avril Zixin Soh, An Pan, Cynthia Bin Eng Chee, Yee-Tang Wang, Jian-Min Yuan and Woon-Puay Koh

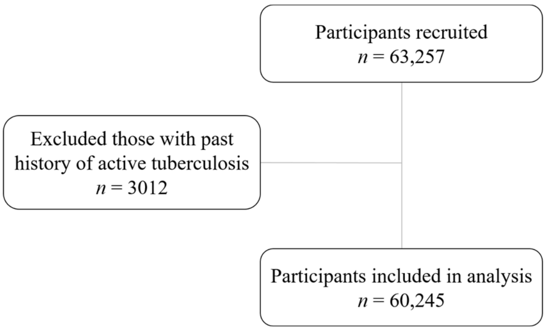

Supplementary Figure S1. Flow diagram of participants included in the final analysis.

Supplementary Table S1. Baseline characteristics of daily tea drinkers by different types of tea [1].

Characteristics	Daily Black Tea Drinkers	Daily Green Tea Drinkers
No. of participants (%)	6640 (49.5)	7485 (55.8)
Daily black tea drinkers		1271 (17.0)
Daily green tea drinkers	1271 (19.1)	
Age at interview (years)	55.2 ± 7.6	56.9 ± 8.0
Body mass index (kg/m^2)	23.3 ± 3.3	23.7 ± 3.3
Men	4046 (60.9)	4047 (54.1)
Dialect		
Cantonese	2982 (44.9)	4338 (58.0)
Hokkien	3658 (55.1)	3147 (42.0)
Level of education		
No formal education	1161 (17.5)	1414 (18.9)
Primary school (1–6 years)	2832 (42.7)	3480 (46.5)
Secondary school and above	2647 (39.9)	2591 (34.6)
Smoking status		
Never	4235 (63.8)	4891 (65.3)
Former	845 (12.7)	1086 (14.5)
Current	1560 (23.5)	1508 (20.2)
Alcohol intake		
None	5087 (76.6)	5762 (77.0)
Monthly	554 (8.3)	653 (8.7)
Weekly	709 (10.7)	751 (10.0)
Daily	290 (4.4)	319 (4.3)
Baseline history of diabetes	511 (7.7)	891 (11.9)

[1] Data shown are *n* (%) for categorical variables and mean ± SD for continuous variables.

Supplementary Table S2. Baseline characteristics of participants who developed active tuberculosis (TB) and those who remained free of TB [1].

Characteristics	TB Cases (*n* = 1249)	Non-Cases (*n* = 58,996)	*p*-Value
Age at recruitment, years	59.3 ± 7.9	56.3 ± 8.0	<0.001
Body mass index, kg/m^2	22.2 ± 3.5	23.2 ± 3.3	<0.001
Men	915 (73.3)	24,999 (42.4)	<0.001
Dialect group			
Cantonese	539 (43.2)	27,438 (46.5)	
Hokkien	710 (56.9)	31,558 (53.5)	0.02
Level of education			
No formal education	308 (24.7)	16,302 (27.6)	
Primary school (1–6 years)	667 (53.4)	25,849 (43.8)	
Secondary school and above	274 (21.9)	16,845 (28.6)	<0.001
Smoking status			
Never smoker	534 (42.8)	41,898 (71.0)	
Former smoker	175 (14.0)	6262 (10.6)	
Current smoker	540 (43.2)	10,836 (18.4)	<0.001
History of diabetes	175 (14.0)	5226 (8.9)	<0.001
Alcohol intake			
None	944 (75.6)	48,106 (81.5)	
Monthly	92 (7.4)	4237 (7.2)	
Weekly	116 (9.3)	4720 (8.0)	
Daily	97 (7.8)	1933 (3.3)	<0.001

[1] Data shown are *n* (%) for categorical variables and mean ± SD for continuous variables. *p*-value by Student's t-test (continuous variables) or chi-square test (categorical variables).

Supplementary Table S3. Baseline factors in relation to risk of active tuberculosis.

Characteristics	HR (95% CI) [1]	HR (95% CI) [2]
Age at recruitment, years	1.06 (1.06–1.07)	1.06 (1.05–1.06)
Body mass index, kg/m^2		0.92 (0.90–0.93)
Year of recruitment		
1993–1995	1.00	1.00
1996–1998	0.85 (0.75–0.97)	0.86 (0.76–0.98)
Gender		
Men	1.00	1.00
Women	0.24 (0.21–0.27)	0.30 (0.25–0.35)
Dialect group		
Cantonese	1.00	1.00
Hokkien	1.08 (0.96–1.21)	1.01 (0.90–1.13)
Level of education		
No formal		1.00
Primary school (1–6 years)		0.97 (0.84–1.13)
Secondary school and above		0.72 (0.59–0.86)
Smoking status and intensity		
Never		1.00
Former 1–12 cig/day		1.41 (1.10–1.80)
Former 13–22 cig/day		0.92 (0.68–1.25)
Former 23+ cig/day		0.92 (0.68–1.26)
Current 1–12 cig/day		1.87 (1.57–2.23)
Current 13–22 cig/day		2.29 (1.93–2.71)
Current 23+ cig/day		2.55 (2.05–3.17)
Baseline history of diabetes		
No		1.00
Yes		2.22 (1.88–2.61)

Alcohol intake	
None	1.00
Monthly	0.86 (0.69–1.07)
Weekly	0.84 (0.69–1.02)
Daily	1.32 (1.06–1.64)

[1] HR = hazard ratio, CI = confidence interval. Model 1 was adjusted for age at recruitment (years), year of recruitment (1993–1995, 1996–1998), gender, dialect group (Hokkien, Cantonese), tea intake (none, monthly, weekly, daily); [2] Further adjusted for education level (no formal education, primary school, secondary school or higher), body mass index (kg/m^2, continuous) baseline history of diabetes (yes, no), smoking status and intensity (never, former 1–12 cig/day, former 13–22 cig/day, former 23+ cig/day, current 1–12 cig/day, current 13–22 cig/day, current 23+ cig/day), alcohol intake (none, monthly, weekly, daily).
